# Research and Experiments on a Unipolar Capacitive Voltage Sensor

**DOI:** 10.3390/s150820678

**Published:** 2015-08-21

**Authors:** Qiang Zhou, Wei He, Songnong Li, Xingzhe Hou

**Affiliations:** 1 State Key Laboratory of Power Transmission Equipment & System Security and New Technology, Chongqing University, Chongqing 400044, China; E-Mail: weihe2016@126.com; 2 State Grid Chongqing Electric Power Co. Electric Power Research Institute, Chongqing 400015, China; E-Mails: songnongli2016@126.com (S.L.); xinzhehou2016@sina.com (X.H.)

**Keywords:** voltage sensor, differential input, non-contact measurement, Ansoft Maxwell

## Abstract

Voltage sensors are an important part of the electric system. In service, traditional voltage sensors need to directly contact a high-voltage charged body. Sensors involve a large volume, complex insulation structures, and high design costs. Typically an iron core structure is adopted. As a result, ferromagnetic resonance can occur easily during practical application. Moreover, owing to the multilevel capacitor divider, the sensor cannot reflect the changes of measured voltage in time. Based on the electric field coupling principle, this paper designs a new voltage sensor; the unipolar structure design solves many problems of traditional voltage sensors like the great insulation design difficulty and high costs caused by grounding electrodes. A differential signal input structure is adopted for the detection circuit, which effectively restrains the influence of the common-mode interference signal. Through sensor modeling, simulation and calculations, the structural design of the sensor electrode was optimized, miniaturization of the sensor was realized, the voltage division ratio of the sensor was enhanced, and the phase difference of sensor measurement was weakened. The voltage sensor is applied to a single-phase voltage class line of 10 kV for testing. According to the test results, the designed sensor is able to meet the requirements of accurate and real-time measurement for voltage of the charged conductor as well as to provide a new method for electricity larceny prevention and on-line monitoring of the power grid in an electric system. Therefore, it can satisfy the development demands of the smart power grid.

## 1. Introduction

A voltage sensor is an important part of the electric system and the accuracy and rapidity of sensor measurement decides the instantaneity of on-line monitoring of the smart power grid [1]. Potential sensors (PTs) and capacitive voltage sensors (CVTs) are often applied to the current electric system [2,3,4,5,6]. A PT involves a large volume, complex insulation structure, and high design cost. In order to increase the accuracy of sensor detection, an iron core structure is adopted during design. Ferromagnetic resonance can occur easily during practical application, and the sensor cannot reflect the changes of measured voltage in time [7,8,9,10,11]. As for CVTs, the measurement signal at the secondary side of a CVT is gained by way of step-by-step voltage reduction for the voltage at the high-voltage side. The measurement signal obtained with this method possesses a great lag when compared with the signal at the high-voltage side. Moreover, a CVT involves a large voltage as well as complex installation and application [12,13,14]. Therefore, traditional voltage sensors are unsuitable for an intelligent development of the power grid. Meanwhile, a direct electric connection is required with the high-voltage conductor when a traditional voltage sensor is used. Thereby, the application range of voltage sensors is restricted [15].

To address the above problems, a new voltage sensor is designed. Different from traditional voltage sensors, this sensor adopts a unipolar structure design which solves the problems of traditional voltage sensors like great insulation design difficulty and high costs caused by the grounding electrode. A differential signal input structure is adopted for the detection circuit, which effectively restrains the influence of common-mode interference signals [16,17,18]. Through sensor modeling, simulation and calculations, the structural design of the sensor electrode is optimized, miniaturization of the sensor is realized, the voltage division ratio of sensor is enhanced, and the sensor measurement phase difference is weakened.

**Figure 1:**
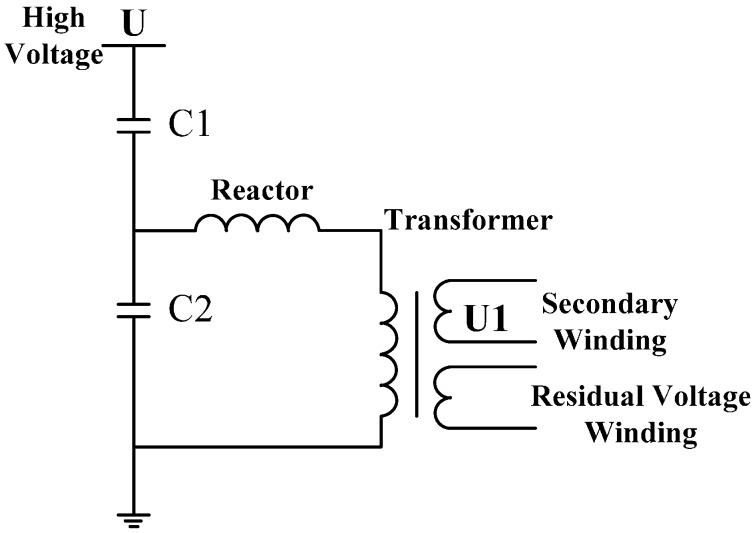
Typical capacitor voltage sensor.

A typical capacitor voltage sensor (CVT) is presented in Figure 1. Its working principle is to divide the voltage U of the conductor to be measured with a voltage division capacitor and to obtain the detection signal U1 at the secondary winding through the sensor.

In other words, a CVT directly measures the voltage of the conductor to be measured using the following equation:

(1) $$U1=\frac{C_{1}}{C_{1}+C_{2}}U$$

Meanwhile, the following requirements should be met when a CVT is used:

(1) One end of the CVT should be connected to the ground;
(2) The CVT must have a direct electrical connection with the high-voltage terminal.

The sensor designed in this paper has essential differences with the CVT, and these differences can be summarized as follows:

(1) By adopting capacitor voltage division principle, CVT conducts direct measurement for the conductor voltage. However, sensor designed by this paper measuring the electric field intensity around the charged conductor. Suppose that the voltage value of conductor is *φ*_(*t*)_, electric field intensity generated by it is *E*_(*t*)_, and time-dependent inductive charge *Q* will be generated at the induction electrode of instrument sensor owing to electric field coupling principle. Through formula derivation, the following relation is gained:
  (2) $$\varphi_{\left(t\right)}=\frac{r^{2}}{\varepsilon_{0}AR_{m}r_{1}}\int \text{​}V_{\left(t\right)}dt$$
  (3) $$V_{\left(t\right)}=R_{m}\frac{dq}{dt}=R_{m}\underset{\text{A}}{\oint }\varepsilon_{0}\frac{dE_{\left(t\right)}}{dt}dA$$

According to Equations (2) and (3), the voltage of the charged conductor can be measured indirectly by measuring the electric field intensity around the conductor to be measured. This is different from the CVT voltage measurement method.

(2) Differing from a CVT in voltage measurement, the instrument sensor designed in this paper does not have electrical connections with the high-voltage side when in use.

The voltage sensor is applied to a single-phase voltage class line of 10 kV for testing. According to the test results, the designed sensor is able to meet the requirements of accurate and real-time voltage measurement as well provide a new method for electricity larceny prevention and on-line monitoring of the power grid in smart power grids, therefore, it can satisfy the development demands of smart power grids.

## 2. Design Principle of the Voltage Sensor

The voltage sensor introduced in this paper is designed on the basis of the electric field coupling principle. Its essence is the principle that electric charges are distributed on the conductor and the electric potential of the conductor involves the relation between conductor and ground, so the electric potential of the conductor also presents a linear relation with electric field intensity around the conductor. By introducing a sensor into the periphery of conductor to be measured, a signal directly proportional to the differential of the electric potential of conductor to be measured over time is obtained, and this signal is integrated, so as to obtain the electric potential value of the conductor. The difference between this result and earth potential is the voltage value of conductor to be measured.

**Figure 2:**
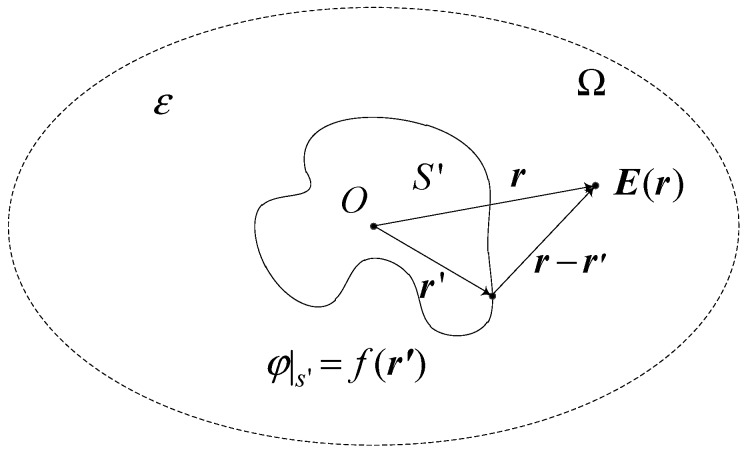
Calculation for the electric field distribution of a charged body.

As shown in Figure 2, *S* is the outer surface boundary of charged conductor of any shape; Ω indicates the calculation region; *ε* represents the electric medium constant in the region; Ω meets asymptotic boundary conditions [19].

The potential distribution on the outer surface of charged conductor is *f*(*r*'); *r'* is the position vector at the original point, *r* represents the position vector at the calculation site; *E*(*r*) indicates the electric field intensity. There is no free charge in the calculation region, which means *ρ*(*r'*) = 0. In other words, the electric potential in the calculation region fulfills the Poisson equation:

(4) $$\nabla^{2}\varphi_{\left(r\right)}=-\frac{\rho \left(r\right)}{\varepsilon }$$

By introducing the Green function, Equation (4) is obtained according to *ρ*(*r'*) = 0 and the first kind of boundary condition, *φ/s'* = *f*(*r'*) [20]:

(5) $$\varphi_{0}=\frac{1}{\varepsilon F(r)}E(r)$$

In the equation, *F*(*r*) is determined by the position of charged body at the site. According to Equation (5), for any point in the calculation region, the electric field intensity is always in direct proportion to the electric potential of the conductor. Moreover, they present a linear relation.

**Figure 3:**
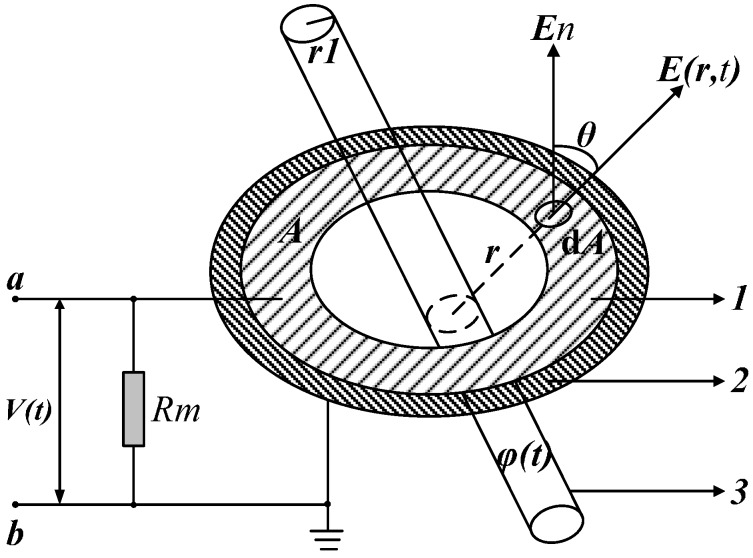
Measurement principle of electric field coupling sensor.

Figure 3 shows the measurement principle of the voltage sensor based on the electric field coupling principle. Label 1 is a metal electrode of any shape and its outer surface is A. Label 2 represents the insulator; as the support of metal electrode, it is connected to the ground. Label 3 indicates the energized cylindrical conductor; its bottom radius is *r_1_* and its electric potential is *φ*(*t*). The metal electrode is connected to the sampling resistance Rm through a coaxial cable and the other side of the resistance is connected to the ground.

The electric field generated by the conductor charge in the surrounding space is *E*(*r*,*t*). Under the effect of the electric field, an inductive charge will appear on the surface of the metal electrode owing to electrostatic induction principle [20,21]. Suppose that the quantity of electric charge is *q*. A closed Gaussian surface is created on the surface of the metal electrode and the infinitesimal *dA* is taken. Its angular separation with the electric field *E*(*r*,*t*) in the normal direction is *θ*. The following equation can be obtained from the Gauss theorem:

(6) $$\frac{dq}{dt}=\oint_{\text{A}}\varepsilon_{0}\frac{\text{d}E(r,t)}{dt}dA$$

According to Equation (6), the electric field intensity changes with time, and the inductive charge varies accordingly. At this time, voltage drop in resistance *R_m_* is:

(7) $$V_{\left(t\right)}=R_{m}\oint_{\text{A}}\varepsilon_{0}\frac{\text{d}E(r,t)}{dt}dA=\frac{\varepsilon_{0}AR_{m}r_{1}}{r^{2}}\frac{d}{dt}\;\varphi \left(t\right)$$

Therefore, the voltage of the charged conductor is:

(8) $$\varphi \left(t\right)=\frac{r^{2}}{\varepsilon_{0}AR_{m}r_{1}}\int \text{​}V\left(t\right)dt$$

According to Equation (8), by integrating the output signal *V*(*t*) of sensor, the voltage in the conductor can be obtained.

## 3. Sensor Structure Design

### 3.1. Structure of the Sensor

**Figure 4:**
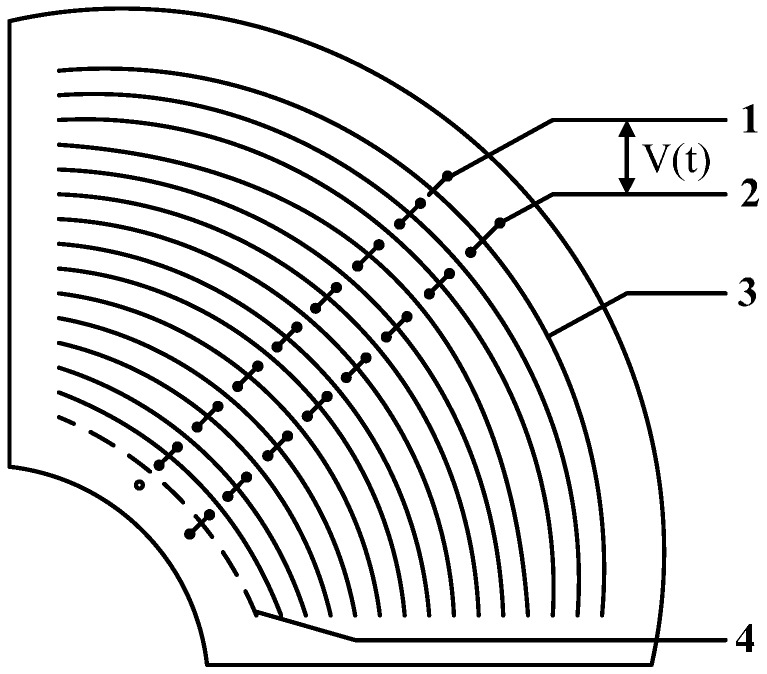
Structure of voltage sensor. 1. Electrode 1; 2. Electrode 2; 3. Annular electrode on the positive side; 4. Annular electrode on the reverse side.

Figure 4 shows the structure of the voltage sensor based on the electric field coupling principle. Annular metal electrodes are etched on both sides of a printed-circuit board (PCB) as induced electrodes. Electrodes of even number on the positive side of the PCB and electrodes of odd number on the reverse side are connected via the hole to form Electrode 1 of the sensor detection electrode. Electrodes of odd number on the positive side of the PCB and electrodes of even number on the reverse side are connected via the hole to form Electrode 2 of the sensor detection electrode. Electrodes connected together have the same electric potential. The sensor can be composed of several PCBs joined together. Electrode 1 of the annular electrodes on each PCB will be connected together, and similarly Electrode 2 on each PCB will be connected together. 

A unipolar design is adopted for the sensor. In other words, the induced electrode of the sensor is earth-free and the potential difference *V*(*t*) between the annular electrodes is used as the signal output of the sensor. A differential amplifier is introduced through a coaxial cable to obtain the difference between two input signals and perform amplification. Such a design solves the problems of insulation design difficulty caused by the grounding electrode of the sensor. Meanwhile, it greatly reduces the insulation design cost and difficulty.

### 3.2. Amplitude-Frequency Characteristics of the Electric Field Coupling Sensor

**Figure 5:**
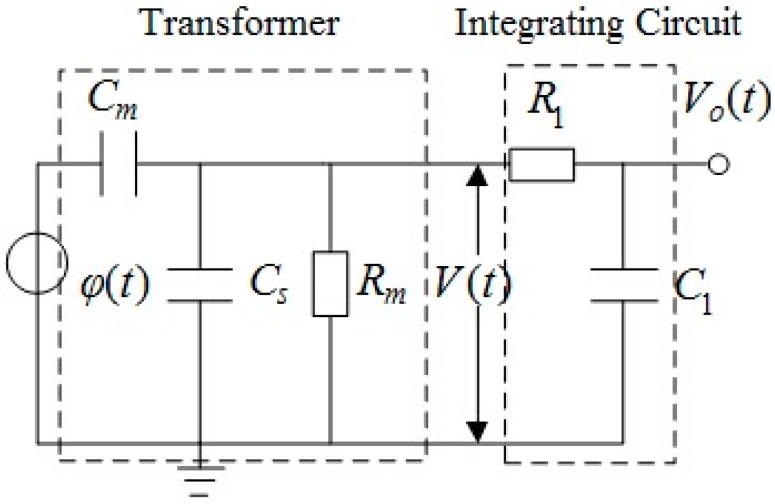
Equivalent circuit of the probe and passive integrator.

The measurement signal output by the instrument sensor is a microcomponent directly proportional to the intensity value of the electric field to be measured. When the influence of frequency change is considered, an equivalent distributed capacitance exists among nearby and insulated conductors due to the difference of electric potential. Therefore, the lumped parameter equivalent circuit model of the entire measurement system presented in Figure 5 can be used.

The voltage of the conductor to be measured is equivalent to the voltage source whose potential to ground is *φ*(*t*). *C_m_* is the equivalent distributed mutual capacitance between the sensor electrode and the conductor to be measured, *C_s_* refers to the equivalent distributed stray capacitance to ground of the sensor electrode, and *R_m_* means the grounding load resistance. The sensor is equivalent to a first-order RC circuit composed of *C_m_*, *C_s_* and *R_m_*. The sensor outputs *V*(*t*) to a passive integral circuit composed of resistor *R_1_* and capacitor *C_1_*, and the output signal *V_o_*(*t*) of the integral circuit is the obtained measurement signal.

The transfer function of sensor can be obtained through a Laplace transformation:

(9) $$H_{p}\left(s\right)=\frac{V\left(s\right)}{\varphi \left(s\right)}=\frac{C_{m}R_{m}s}{\left(C_{m}+C_{S}\right)R_{m}s+1}$$

The transfer function of passive integral circuit is:

(10) $$H_{I}\left(s\right)=\frac{V_{0}\left(s\right)}{V\left(s\right)}\frac{1}{C_{1}R_{1}s+1}$$

**Figure 6:**
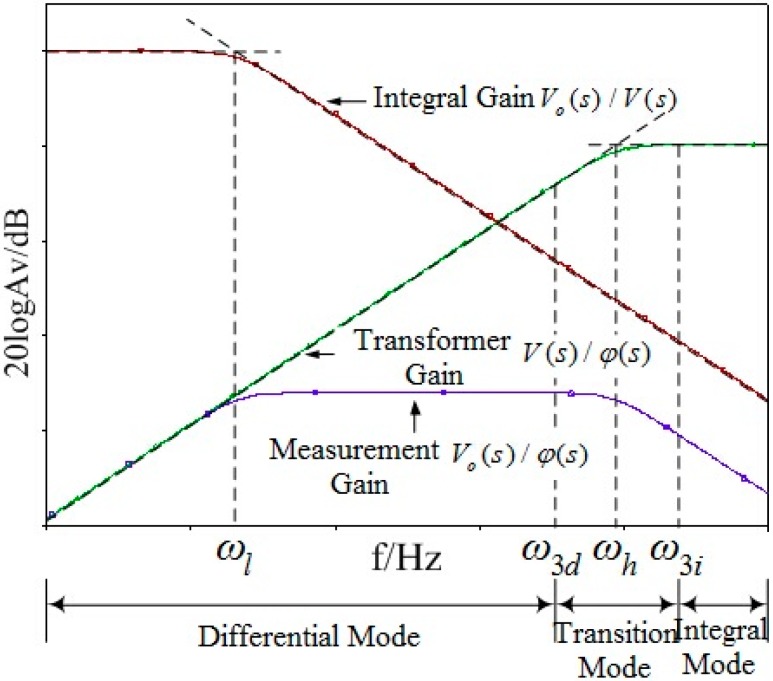
Frequency response of the equivalent circuit.

The characteristic amplitude-frequency response curve of the sensor, integral circuit and the entire measurement system can be obtained through Equations (9) and (10), as shown in Figure 6:

In Figure 6, *ω_l_* and *ω_h_* are the corner frequencies of the amplitude-frequency characteristic curves of the passive integral circuit and electric field coupling sensor, and meanwhile they are also the lower frequency limit and upper frequency limit of the entire measurement system. They will decide the measurement bandwidth of the measurement system. *ω_3d_* and *ω_3i_* are the frequency points of decreasing and increasing the sensor gain by 3 dB from the corner frequency *ω_h_* of the sensor:

(11) $$\omega_{l}=\frac{\text{1}}{C_{1}R_{1}}$$

(12) $$\omega_{h}=\frac{1}{\left(C_{m}+C_{S}\right)R_{m}}$$

When (*C_m_* + *C_s_*) *R_m_*>>1 (the lower frequency limit of the measured voltage *ω_a_*>*ω_3i_*), the transfer function of the sensor can be obtained according to Equation (12):

(13) $$H_{p}\left(s\right)=\frac{V\left(s\right)}{\varphi \left(s\right)}=\frac{C_{m}}{C_{m}+C_{s}}$$

In such a situation, *V*(*s*) and *φ*(*s*) are directly proportional, and the sensor operates in self-integral mode. The sensor is able to make its input and output present a linear relation without an integral circuit. Generally speaking, the voltage frequency in the electrical power system is within the fundamental frequency range of 50 Hz, but the transient voltage frequency might reach a level of MHz. The sensor needs to maintain a stable gain in such a wide frequency range and increase the frequency range as much as possible, so a sensor operating in self-integral mode has more advantages when applied to voltage measurement of electrical power systems by considering a series of problems in the integral circuit. When designing the sensor in this paper, a sensor operating in self-integral mode will be used, so as to make it suitable for application in electrical power systems.

### 3.3. Differential Input Structure

In order to make electric field coupling voltage sensor operate in self-integral mode under the power frequency of 50 Hz, and realize the purposes of expanding bandwidth, reducing error and avoiding the influence of the integral circuit, the corner frequency *ω_h_* should at least meet the following conditions:

(14) $$\omega_{h}=\frac{1}{\left(C_{m}+C_{S}\right)R_{m}}<2\pi \times 50$$

Therefore, the value of (*C_m_* + *C_s_*)*R_m_* should at least meet the following conditions:

(15) $$\left(C_{m}+C_{S}\right)R_{m}>3.18\times 10^{-3}$$

Only in this way, can the electric field coupling sensor operate in self-integral mode. In view of the insulating strength and geometrical parameters and structures of the conductor, the distance between the electric field coupling sensor and the measured conductor should not be too close, and the capacitance value of the mutual capacitor *C_m_* between the measured conductor and sensor electrode and the capacitance value of the stray capacitor to the ground *C_s_* of the electrode are of pF (10^−12^) magnitude. In order to meet the conditions in Equation (15), a load resistor *R_m_* with a resistance value of GΩ grade should be used. Thus problems exist in resistor selection and impedance matching. If the resistance value is too small, the corner frequency of the transfer function will move toward a high-frequency region, which can reduce the measurement bandwidth.

**Figure 7:**
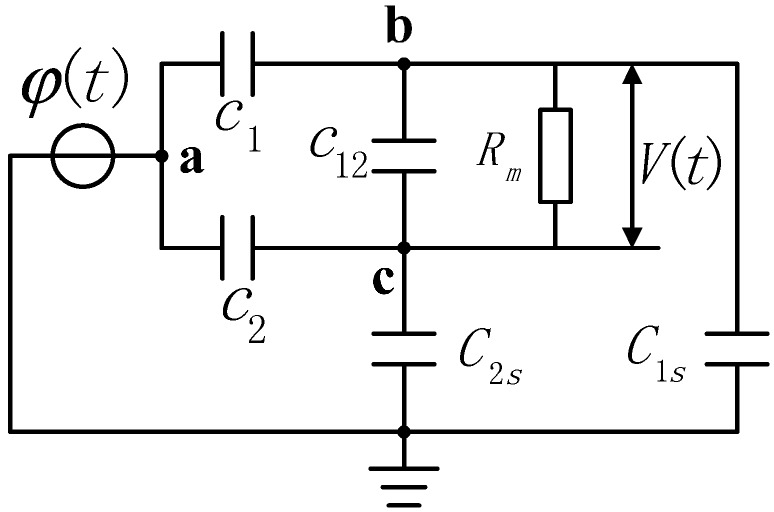
Equivalent circuit of the voltage sensor.

In order to solve the above problems, an electric field coupling sensor of differential input structure is proposed in this paper. Figure 7 shows the equivalent circuit of the voltage sensor. In this figure a is the electric potential of the conductor to be measured, and mode b and c are floating electrode potentials. The potential difference *V*(*t*) is the input signal of the differential amplifier, *C*_1_ refers to the equivalent distributed capacitance of the conductor to be measured and Electrode 1, *C*_2_ indicates the distributed capacitance of the conductor to be measured and Electrode 2, *C*_12_ denotes the mutual capacitance between two electrodes, *C*_1*s*_ means stray capacitance to earth of Electrode 1, *C*_2*s*_ represents stray capacitance to earth of Electrode 2, and *R_m_* is the input impedance of the differential amplifier.

According to the circuit structure in Figure 7, the transfer function of the voltage sensor is obtained:

(16) $$H(s)=\frac{V(s)}{\varphi (s)}\frac{\left(C_{1}C_{2s}-C_{2}C_{1s}\right)R_{m}S}{R_{m}S(C_{1}+C_{1s})(C_{2}+C_{2s})+\left(C_{12}R_{m}S+1\right)\left(C_{1}+C_{2}+C_{1s}+C_{2s}\right)}$$

**Figure 8:**
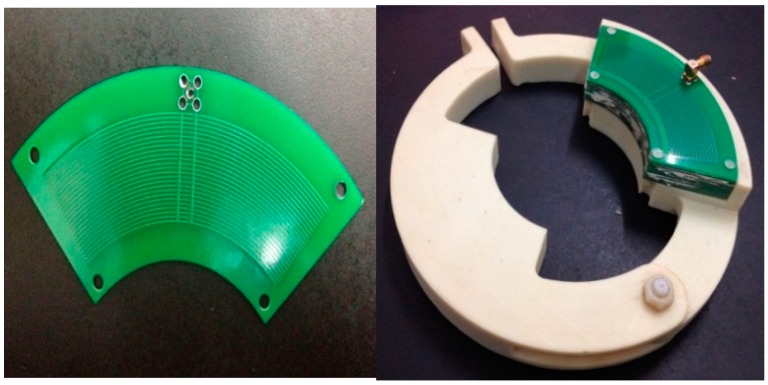
Real sensor appearance.

According to Equation (16), the distance between electrodes can be adjusted freely. Compared with the situation where the mutual capacitance *C*_1_ and *C*_2_ between the conductor to be measured and electrodes cannot acquire a great capacitance value due to the long distance, the mutual capacitance *C*_12_ between electrodes is able to reach a capacitance value far greater than the mutual capacitance between conductor and electrodes as well as the stray capacitance to earth of the electrodes. The increase of the *C*_12_ value will enhance the measurement bandwidth of the sensor, reduce the measurement phase error, and make the sensor work in a self-integral state under low frequency. Therefore, the parallel connection of multiple electrodes shown in Figure 4 is adopted, and multiple sensors are used in superposition. In this way, the value of the mutual capacitance *C*_12_ between electrodes is increased to the largest extent. Figure 8 shows the actual sensor.

## 4. Influence of External Factors

In the sensor designed by this paper, the induction electrode is designed on the printed circuit board, and the external environment mainly includes the working temperature of the sensor instrument, humidity and the external electromagnetic field.

(1) The change of temperature will affect the equivalent resistance of the induction electrode of the instrument sensor. However, in our case induction electrode is made of copper which is not a heat-sensitive material, so by combining with the temperature coefficient of copper within the scope of 0–100 °C, the resistance change of the sensor designed by this paper with temperature can be ignored. Therefore, the influence of temperature on the use of this instrument sensor can be neglected.
(2) During actual use, a shell will be designed for the sensor and the shell is made of nylon plastic. Therefore, moisture condensation will not happen on the surface of the detection electrode due to humidity changes, so the influence of external humidity on the use of this instrument sensor can be ignored too.
(3) For power frequency electric field detection, the electric field coupling principle is used to design this sensor. Besides, a suspended potential differential input mode is adopted for the instrument sensor, and a loop is formed. In terms of external electric field, the signal generated by a high-frequency electric field is a high-frequency signal, and the follow-up processing circuit of the sensor adopts a band-pass filter which can effectively filter out high-frequency components. At the same time, the differential input mode is able to effectively filter out common-mode voltage signals.

The external power frequency voltage signal has a huge influence on this sensor instrument, but the influence of the external power frequency voltage signal can be reduced by decreasing the sensor size, keeping it away from charged bodies, and adopting a symmetric design for the induction electrode.

## 5. Simulations

**Figure 9:**
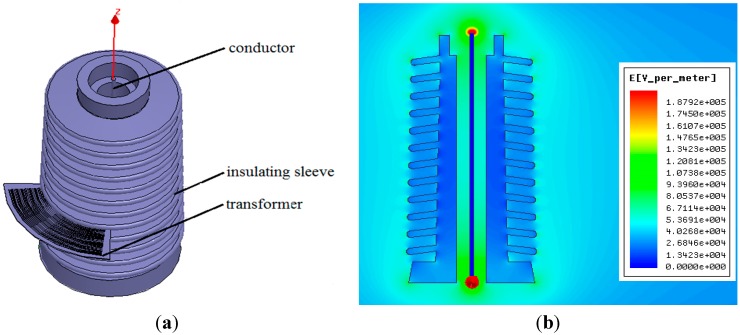
Simulation model. (**a**) Calculation model; (**b**) Electric field intensity distribution (cross-section).

In experiments, the above voltage sensor was put in an insulating sleeve. Therefore, modeling and simulation were conducted for the insulating sleeve of the sensor, conductor and voltage sensor in the electromagnetic field finite element software Ansoft Maxwell environment. The degree of distortion of the electric field to be measured after introducing sensor was analyzed by comparison. Meanwhile, simulation calculations were carried out to verify whether the structural design of the sensor meets the insulation design requirements and the relationship between structural design of the sensor and inter-electrode capacitance. Figure 9 is the simulation model, in which 1 is the conducting rod in the insulating sleeve of the sensor, 2 represents the insulating sleeve, and 3 refers to the voltage sensor. During the simulation, it was thought that the electric field around the insulating sleeve was only generated by the electric charge on the conducting rod in the insulating sleeve, without interference from any external electric field. In practical experiments, other charged bodies were kept away from the electric field to be measured, so as to ensure an experimental environment with little interference [22].

### 5.1. Distortion of the Electric Field to be Measured Caused by the Voltage Sensor

**Figure 10:**
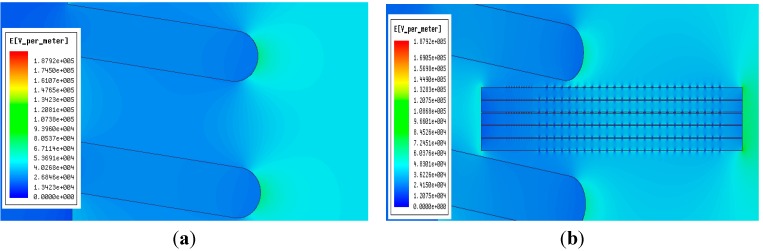
Influence of the voltage sensor on the electric field to be measured. (**a**) Electric field distribution before introducing the voltage sensor; (**b**) Electric field distribution after introducing the voltage sensor.

During simulation, the conductor voltage in the insulating sleeve is set as 10 kV. After the simulation, Figure 10a shows the electric field distribution inside and outside the insulating sleeve before introducing the voltage sensor. From the simulation, we can see that in the insulating sleeve, near the end of the conductor, the electric field strength is about 250 kV/m, and the electric field intensity close to the internal side and external side of the insulating sleeve is about 65 kV/m. The greater the distance to the insulating sleeve is, the lower the electric field intensity will be. Figure 10b presents the influence on the electric field to be measured after introducing voltage sensor. According to the figure, the electric field intensity in the place of the sensor is weakened, while the electric field intensity at the edge of sensor is increased. The electric field intensity distribution around the sensor is even. According to the analysis, introduction of the sensor has resulted in a distortion of the electric field to be measured, but the electric field intensity just declines from 65 kV/m before introducing the sensor to about 48 kV/m. Such a distortion effect will not bring about a huge interference in the measurements of the voltage sensor.

**Table 1:** Calculated value of electric field intensity for different voltage values.

Voltage (kV)	Value of Electric Field Before Using the Sensor (V/m)	Value of Electric Field After Using the Sensor (V/m)
1	4904.36	4127.17
2	9808.00	8254.36
3	14,712.61	12,379.87
4	19,768.98	16,507.49
5	24,520.10	20,633.19
6	29,423.23	24,760.02
7	34,329.97	28,886.87
8	39,232.58	33,014.99
9	44,136.06	37,139.03
10	49,040.67	41,266.37

During the simulation, a point is set up on the upward side of the voltage sensor. The electric field intensity change of this point before and after introducing the voltage sensor is calculated. Table 1 shows the electric field intensity values of this point for different voltage values. 

**Figure 11:**
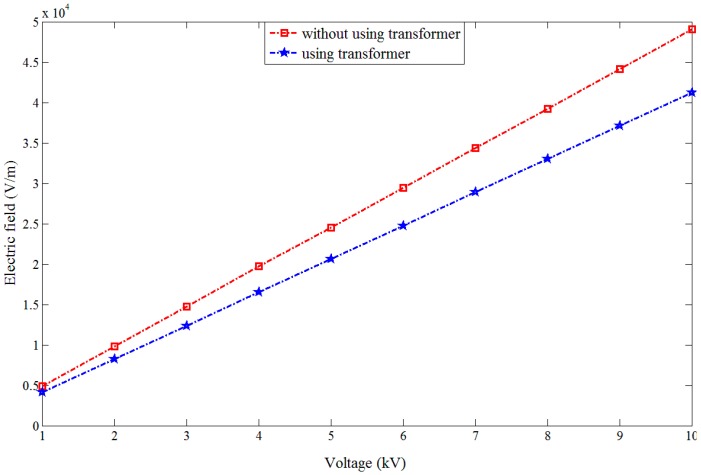
Change of electric field intensity with voltage value.

Figure 11 presents the curve relating the electric field intensity change and voltage value. According to the figure, before introducing the voltage sensor, the electric field intensity of this point presents a linear increasing trend with the voltage increase; after introducing the voltage sensor, a distortion effect is caused to the original electric field and the electric field intensity of the test point is reduced, but it still presents a linear increasing trend as the voltage increases. Therefore, after introducing the voltage sensor designed in this paper, the electric field intensity near the sensor still maintains the relationship whereby the electric field intensity presents a linear variation with voltage change. Moreover, the sensor output signal also shows a linear relation with electric field intensity. Thus the correctness of voltage sensor design principle is verified.

### 5.2. Relationship between the Structural Design of the Sensor and the Inter-Electrode Capacitance

Through follow-up processing of the simulation results, the equivalent distributed capacitance values under different incentive structures and electrode structures can be obtained. Thus the sensor structure design can be optimized.

**Figure 12:**
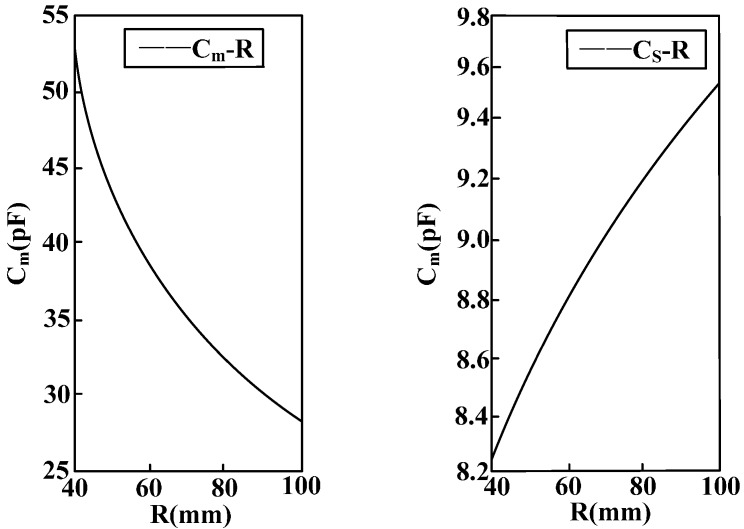
Curve of the relation between equivalent capacitance parameters and electrode radius.

Figure 12 shows the relation between sensor radius R and equivalent capacitance *C_m_* between electrodes as well as stray capacitance to earth *C_s_* of the electrodes. When the sensor electrode radius R increases, the equivalent capacitance *C_m_* between electrodes is reduced and the stray capacitance to earth *C_s_* of the electrodes increases. Moreover, the amount of change of the equivalent capacitance between electrodes is far greater than the amount of change of the stray capacitance to earth of the electrodes.

**Figure 13:**
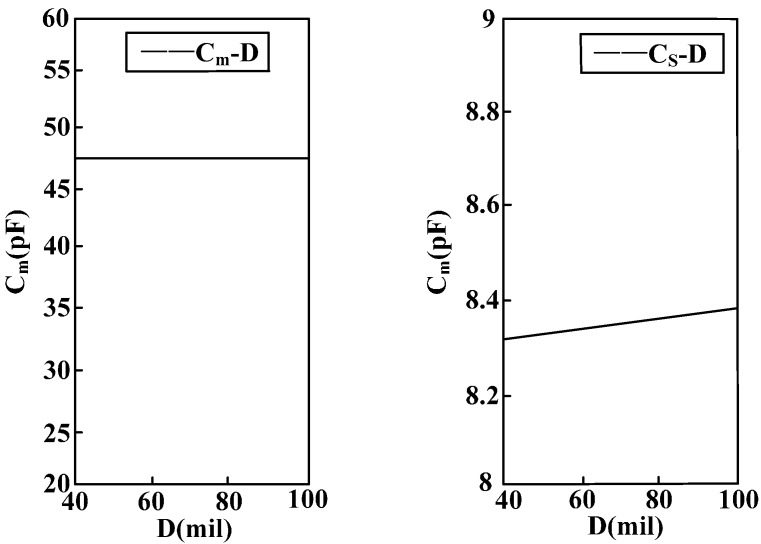
Curve of the relation between equivalent capacitance and electrode width.

Figure 13 shows the relation between sensor electrode width D and equivalent capacitance *C_m_* between electrodes as well as stray capacitance to earth *C_s_* of the electrodes. When the sensor electrode width increases, the equivalent capacitance *C_m_* between electrodes remains unchanged and stray capacitance to earth *C_s_* of electrodes increases with the increase of electrode width.

**Figure 14:**
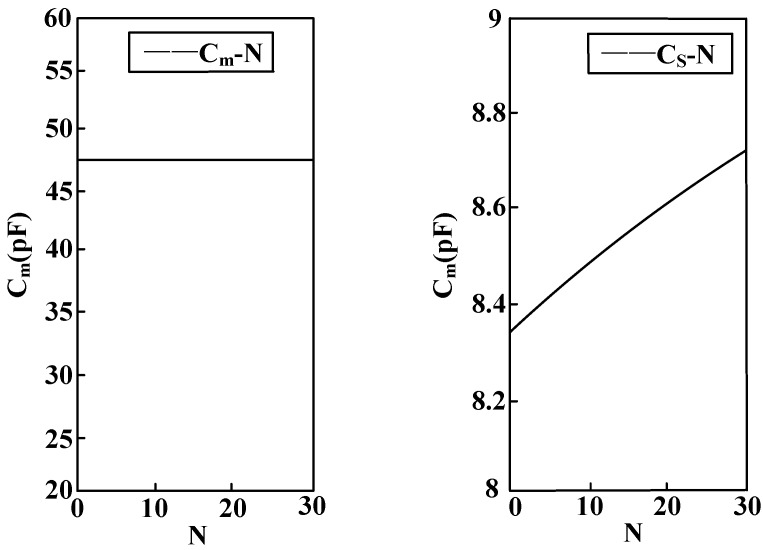
Curve of the relation between equivalent capacitance and number of electrodes.

Figure 14 shows the relation between number N of parallel-connection electrodes of the sensor and equivalent capacitance *C_m_* between electrodes as well as stray capacitance to earth *C_s_* of the electrodes. When the number of parallel-connection electrodes of the sensor rises, the equivalent capacitance *C_m_* between electrodes remains unchanged and stray capacitance to earth *C_s_* of electrodes increases with the increasing number of electrodes.

According to the above analysis, it is known that in order to restrain the measurement error disturbance caused by the stray capacitance to earth of the electrodes, the electrode radius should be reduced, and the electrode width and distance between electrodes must be decreased on the premise of guaranteeing electrode insulation. In this way, more electrodes can be arranged in a small space and equivalent capacitance between electrodes will be increased to the largest extent possible. Thereby, voltage division ratio and measurement accuracy can be increased, and phase difference will be reduced.

### 5.3. Insulation Design of Sensor

If the electric field intensity borne by the dielectric medium used as insulating material on the sensor is higher than the critical electric field intensity of this medium, it will lose insulating ability and be damaged. Thus the insulating property will disappear, and partial discharges or short circuits between electrodes can occur. Therefore, the maximum electric field intensity distribution in the internal part and at the boundary of the sensor during application should be calculated, and the design of the sensor must be perfected to account for this. It has to possess good insulating properties under the stipulated voltage rating, and some surplus capacity must be reserved.

**Figure 15:**
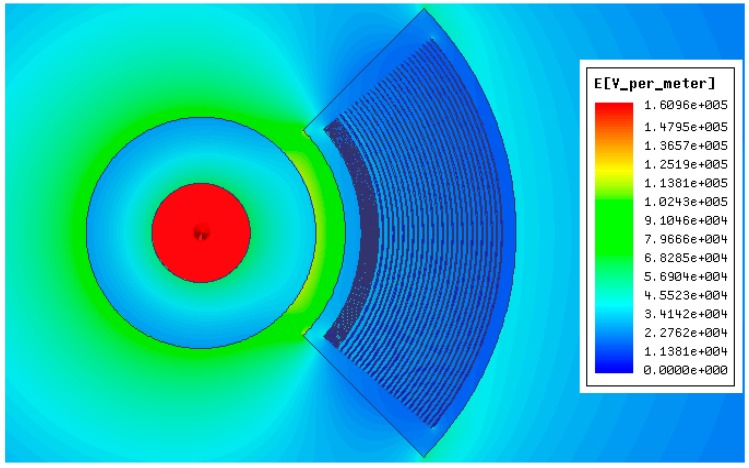
Electric field distribution diagram.

**Figure 16:**
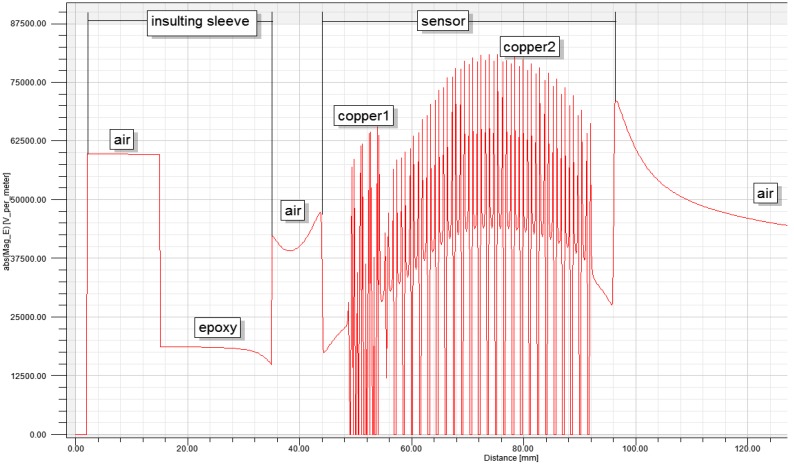
Distribution diagram of the electric field intensity in different media.

In the simulation calculations, the voltage of the conducting rod in the sensor was set as 10 kV. On the established model interface, it can be treated as a multi-dielectric coaxial cylindrical model composed of a guide rod, insulating sleeve, sensor and air. According to the relation between electric field intensity and boundary electric potential shown in Figure 15 and Figure 16, the distribution of the electric field intensity in different dielectric media on the model section is obtained through calculation. Table 2 shows the parameters of multiple media and the critical electric field intensity.

According to the contrastive analysis provided in Figure 15 and Figure 16, Table 2, under s power frequency electric field, the conducting rod is an equipotential body, the internal electric field intensity of the electrode is approximately 0, and the electric field intensity between the is at a low level. A strong electric field intensity is concentrated between the electrodes of the sensor and the highest electric field intensity is 8 kV/m, far lower than the critical electric field intensity of epoxy resin, 200 kV/cm as given in Table 2. Therefore, sensor design completely meets the insulation design requirements.

**Table 2:** Medium parameter.

Medium	Dielectric Coefficient	Critical Electric Field Intensity (kV/cm)
Epoxy	3.6	200–300
Air	1	25–30

## 6. Steady State Test Experiments

**Figure 17:**
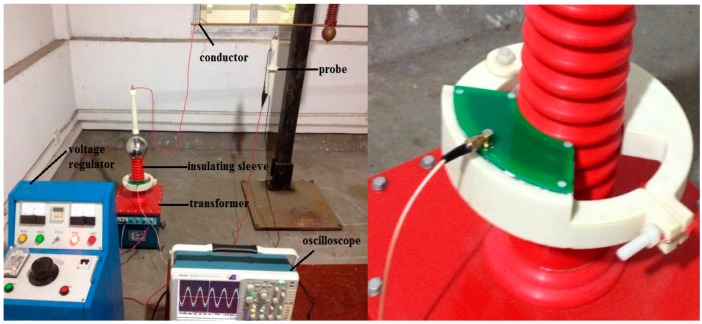
Experimental platform.

In order to verify the performance of the designed instrument transformer, an experimental platform is established, as shown in Figure 17.

The platform is composed of a voltage regulator, transformer and single-phase wire. The voltage regulator has an input voltage of 220 V, output voltage of 0–200 V, and capacity of 50 kV/A, and it is single-phase. As for the transformer, the low voltage of its rated voltage is 200 V, the high voltage is 50 kV, the rated capacity is 10 kV/A, and it is single-phase. The voltage output range that can be realized by the voltage console composed of voltage regulator and transformer is 0–50 kV.

The P6051A high-voltage probe is a capacitance probe, and its compensation range is 7–49 pF. The bandwidth is 75 MHz and attenuation multiple is 1000. Within the measurement scope of 10 kV, its measurement accuracy for AC voltage is lower than 1.5%. The high-voltage probe is connected to the wire, and the output of the high-voltage probe is equal to the voltage value of the charged body in the insulating bush. 

The oscilloscope used in the experiment is a Tektronix MOS5014B; the bandwidth is 1 GHz, it displays six significant digits, the vertical resolution is 11 digits, and the input sensitivity scope is 1 mV/div.

**Figure 18:**
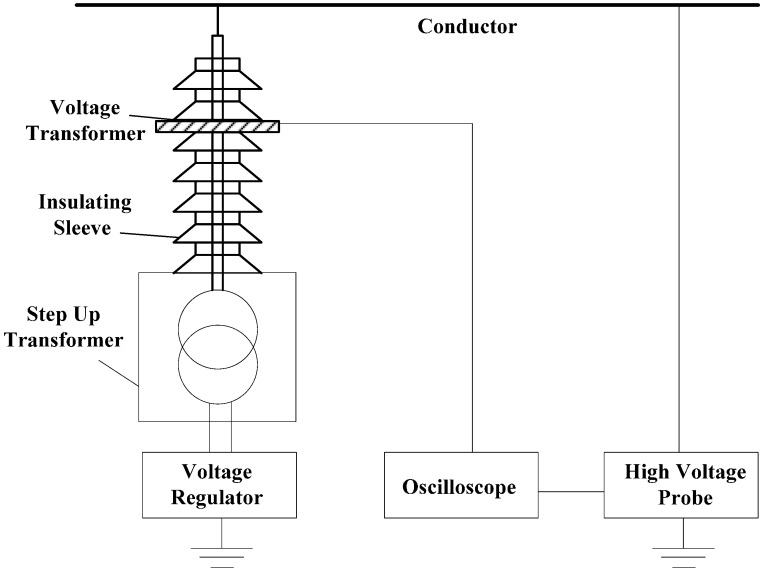
Experiment platform diagram.

During the experiment, as shown in Figure 18, the voltage regulator and transformer form the control part of voltage output, and the conducting rod of the transformer is connected to the conductor. The instrument transformer is stuck in the external part of insulating bush, and the high-voltage probe is suspended above the conductor. The output results of instrument transformer are compared with the output results of the high-voltage probe. It is verified that the output of the instrument transformer designed in this paper is in direct proportion to the conductor voltage. Besides, an experiment is made to verify whether the instrument transformer meets the expected voltage division ratio. 

In the experiment, the sensor output voltages from 0 kV to 10 kV through the voltage regulator; signal waveforms of sensor and high-voltage probe under currents of 1, 2, 3, 4, 5, 6, 7, 8, 9 and 10 kV were collected and recorded. Moreover, the ratio error *ε*% and phase difference *φ* between the sensor signal and actual voltage signal were calculated according to the stipulations in the IEC60044-7 standard. The definitions are as follows:

(17) $$\varepsilon \text{\%}=\frac{KU_{S}-U_{H}}{U_{H}}\times \text{100\%}$$

(18) $$\varphi =\varphi_{S}-\varphi_{H}$$

In the formula, K is the rated transformation ratio of sensor and rated transformation ratio of sensor is 2000:1 after calibration. *U_S_* refers to the measurement signal voltage output by the voltage transducer, and *U_H_* means the primary voltage measured by the voltage transducer, which is the value of converting the output voltage measured by the high-voltage probe as a standard apparatus into the primary side. The transformation ratio of the high-voltage probe is 1000:1. *φ_S_* is the output voltage phase of the voltage transducer and *φ_H_* means the output voltage phase of the high-voltage probe. See Table 3 for the experimental data.

According to Table 3, within the voltage range of 10 kV, the voltage ratio error of the voltage sensor designed on the basis of the electric field coupling principle *ε*% < 0.5%, and phase difference *φ* < 30′. Therefore, the measurement accuracy of such a voltage sensor under steady state conditions meets the requirements of actual measurement.

**Table 3:** Measurement accuracy results of the voltage sensor.

Voltage (kV)	*U_H_* (kV)	*U_S_* (kV)	*ε*/%	*φ*/(')
1	0.975	0.489	0.23	35
2	2.013	1.001	0.20	31
3	3.051	1.510	0.15	27
4	3.977	1.990	0.09	25
5	5.014	2.504	−0.11	19
6	5.965	2.990	−0.25	22
7	7.109	3.543	−0.31	17
8	8.071	4.025	−0.27	16
9	8.986	4.479	−0.32	21
10	9.951	4.963	−0.26	30

**Figure 19:**
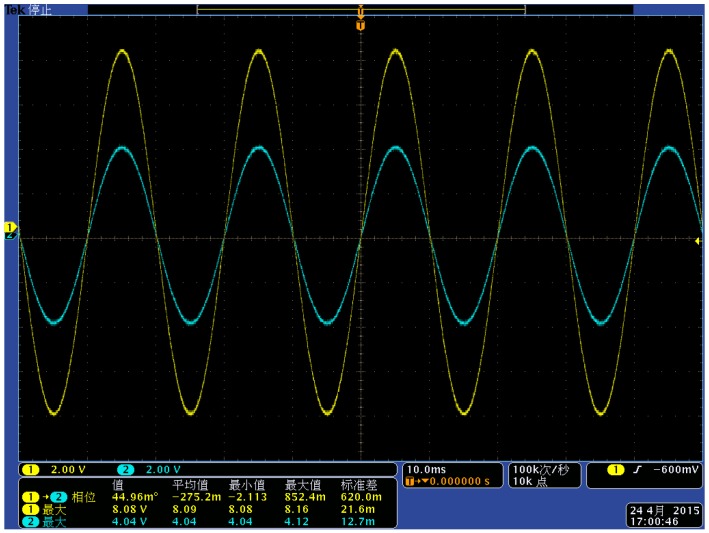
Comparison diagram of waveform under the voltage class of 2 kV.

**Figure 20:**
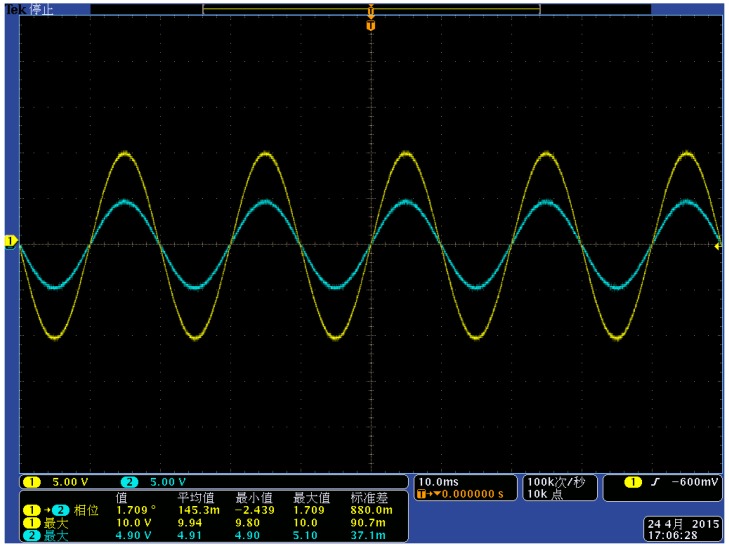
Comparison diagram of waveform under the voltage class of 8 kV.

Figure 19 and Figure 20 show the waveforms collected by the oscilloscope when the line voltage is 8 kV and 10 kV. Channel 1 is the signal waveform of the voltage sensor (yellow waveform) and channel 2 is the signal waveform of the high-voltage probe (blue waveform). According to Figure 19 and Figure 20, the sensor works in a self-integral state and the detection signal can follow the changes of the actual voltage waveform. The phase difference is small and the linearity is good. The following conclusions can be put forth according to Table 3, Figure 19 and Figure 20:

(1) The measurement accuracy of the voltage sensor designed in this paper under steady state conditions is high and the phase error with the actual voltage is small. Therefore, it can meet the measurement requirements;
(2) The structural design of the voltage sensor can realize the situation where the sensor works in a self-integral state; the voltage division ratio reaches the design requirement of 2000:1;
(3) The experiments prove that the designed voltage sensor possesses good linearity, and it can realize non-contact detection for conductor voltages by measuring the electric field intensity around a charged conductor.

## 7. Conclusions and Outlook

In this paper, a new voltage sensor based on the electric field coupling principle is designed. According to simulations and experiments, the designed voltage sensor has excellent steady-state performance. Under high voltage loads, the sensor is able to meet the requirements of insulation design. Moreover, non-contact detection for charged conductor voltage can be realized and the application range of the sensor is expanded through the open design. Meanwhile, the unipolar design has solved the insulation design difficulty problem of voltage sensors, and therefore, it can satisfy the development demands of the smart power grid.
